# Obesity and Associated Factors — Kingdom of Saudi Arabia, 2013

**DOI:** 10.5888/pcd11.140236

**Published:** 2014-10-09

**Authors:** Ziad A. Memish, Charbel El Bcheraoui, Marwa Tuffaha, Margaret Robinson, Farah Daoud, Sara Jaber, Sarah Mikhitarian, Mohammed Al Saeedi, Mohammad A. AlMazroa, Ali H. Mokdad, Abdullah A. Al Rabeeah

**Affiliations:** Author Affiliations: Mohammad A. AlMazroa, Mohammed Al Saeedi, Abdullah A. Al Rabeeah, Ministry of Health of the Kingdom of Saudi Arabia, Assadah, Al Murabba Riyadh, Saudi Arabia; Charbel El Bcheraoui, Marwa Tuffaha, Margaret Robinson, Farah Daoud, Sara Jaber, Sarah Mikhitarian, Ali H. Mokdad, Institute for Health Metrics and Evaluation, University of Washington, Seattle, Washington.

## Abstract

**Introduction:**

Data on obesity from the Kingdom of Saudi Arabia (KSA) are nonexistent, making it impossible to determine whether the efforts of the Saudi Ministry of Health are having an effect on obesity trends. To determine obesity prevalence and associated factors in the KSA, we conducted a national survey on chronic diseases and their risk factors.

**Methods:**

We interviewed 10,735 Saudis aged 15 years or older (51.1% women) through a multistage survey. Data on sociodemographic characteristics, health-related habits and behaviors, diet, physical activity, chronic diseases, access to and use of health care, and anthropometric measurements were collected through computer-assisted personal interviews. We first compared sociodemographic factors and body mass index between men and women. Next, we conducted a sex-specific analysis for obesity and its associated factors using backward elimination multivariate logistic regression models. We used SAS 9.3 for the statistical analyses and to account for the complex sampling design.

**Results:**

Of the 10,735 participants evaluated, 28.7% were obese (body mass index ≥30 kg/m^2^). Prevalence of obesity was higher among women (33.5% vs 24.1%). Among men, obesity was associated with marital status, diet, physical activity, diagnoses of diabetes and hypercholesterolemia, and hypertension. Among women, obesity was associated with marital status, education, history of chronic conditions, and hypertension.

**Conclusion:**

Obesity remains strongly associated with diabetes, hypercholesterolemia, and hypertension in the KSA, although the epidemic’s characteristics differ between men and women.

## Introduction

Obesity is a major risk factor for illness and death. It is associated with diabetes, hypertension, hyperlipidemia, obstructive sleep apnea, and osteoarthritis (1). With the increase in life expectancy, obesity is causing more years of disability (2). Hence, the increased cost of obesity and its sequelae will put a strain on the resources of governments and individuals (3).

In many developing societies, the adoption of a Western lifestyle, characterized by decreased physical activity and high caloric intake, is contributing to an alarming epidemiological transition marked by the shift in the leading causes of death from communicable to noncommunicable diseases (4,5). The recognition of the role of elevated body mass index (BMI) in these changes has made obesity a high priority for health authorities around the world (6).

Socially, the perception of obesity has changed over time. Whereas it once was associated with wealth and prosperity for men and with health and reproduction abilities for women, it is now perceived as a health problem and a risk factor for many diseases (7). Women, the poor, and older people are at higher risk of obesity worldwide (8,9).

The *Global Burden of Disease 2010* study found that elevated BMI was the leading risk factor for disability-adjusted life years in the Kingdom of Saudi Arabia (KSA) (10). Previous studies in KSA indicate an increasing trend in the prevalence of obesity. Data from the late 1980s through mid-1990s show a prevalence of obesity averaging about 20%, ranging from as low as 13.1% among men to as high as 26.6% among women. However, all prevalence estimates from 1995 and beyond are above 35% (11–13). The last national survey on obesity in the KSA and its associated risk factors was conducted in 2005 in collaboration with the World Health Organization. Since then, no such surveys have been conducted, making it impossible to determine whether the efforts of the Saudi Ministry of Health (SMOH) are affecting obesity trends. To determine current rates of obesity and associated risk factors and chronic conditions, the SMOH, in collaboration with the Institute for Health Metrics and Evaluation, conducted a large household survey in 2013. In this article, we report the findings from that survey.

## Methods

**Figure 1:**
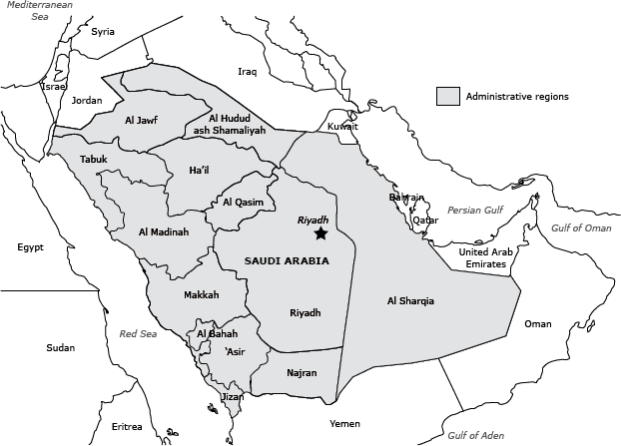
Thirteen administrative health regions, Kingdom of Saudi Arabia.

The Saudi Health Information Survey (SHIS) was a national multistage survey of men and women aged 15 years or older. Households of Saudi citizens were randomly selected from a national sampling frame maintained and updated by the KSA Census Bureau. The Ministry of Health divides KSA into 13 health regions, each with its own health department (Figure 1). For the survey, we divided each region into subregions and blocks used by the KSA department of statistics. All regions were included in the survey, and a probability proportional to size was used to randomly select subregions and blocks. Households were randomly selected from each block. A roster of household members was created, and an adult aged 15 or older was randomly selected from that household to be surveyed. If the randomly selected adult was not present, our surveyors made an appointment to return, and a total of 3 visits were made before the household was considered as a nonresponse. Weight, height, and blood pressure of the randomly selected adult were measured at the household by a trained professional.

The survey included questions on sociodemographic characteristics, tobacco consumption, diet, physical activity, health care use, various health-related behaviors, and self-reported chronic conditions. These conditions were hypertension, diabetes, hypercholesterolemia, stroke, cardiac arrest, myocardial infarction, congestive heart failure, chronic obstructive pulmonary disease, atrial fibrillation, renal failure, asthma, and cancer.

We used measured weight and height to calculate BMI (kg/m^2^). Participants were classified into 4 groups: underweight (BMI <18.5), normal weight (BMI 18.5 –24.9), overweight (BMI 25.0–29.9), or obese (BMI ≥30.0) (14). Respondents were considered to be smokers if they reported currently smoking. We computed the number of servings of fruits, vegetables, red meat, and chicken consumed per day from a detailed dietary questionnaire as the sum of the average daily consumption of fruits, fruit juices, vegetables, red meat, and chicken. We used the International Physical Activity questionnaire (15) to classify respondents into 4 groups of physical activity: 1) vigorous, 2) moderate, 3) insufficient, and 4) none.

To assess diagnosed blood pressure, diabetes, and hypercholesterolemia status, respondents were asked 3 separate questions: “Have you ever been told by a doctor, nurse, or other health professional that you had: 1) hypertension, otherwise known as high blood pressure; 2) diabetes mellitus, otherwise known as diabetes, sugar diabetes, high blood glucose, or high blood sugar; 3) hypercholesterolemia, otherwise known as high or abnormal blood cholesterol?” Women diagnosed with diabetes or hypertension during pregnancy were not counted as having these conditions. Those who reported diagnosis of any of these conditions were asked if they were currently receiving any treatment for their condition. Similarly, the same question format was used to determine previous diagnosis of stroke, myocardial infarction, atrial fibrillation, cardiac arrest, congestive heart failure, chronic obstructive pulmonary disease, asthma, renal failure, and cancer.

Three blood measurements were taken with the participant resting and at 5-minute intervals. We followed the National Health and Nutrition Examination Survey procedures for editing and determining blood pressure levels (16). Briefly, if only 1 reading was available for systolic blood pressure, this reading was used. If 2 readings were available, the second reading was taken into account. If 3 readings were available, the average of the last 2 readings was considered the systolic blood pressure. The same method was used for calculating diastolic blood pressure. Respondents were considered to have elevated blood pressure if they met any of the following criteria: 1) measured diastolic or systolic blood pressure exceeding 90 or 140 mm Hg, respectively, or 2) measured diastolic or systolic blood pressure not exceeding the appropriate threshold but the respondent reporting taking medications for blood pressure.

We compared sociodemographic characteristics and BMI categories between men and women by using a multivariate logistic regression model. We used a backward elimination multivariate logistic regression model to measure association between obesity and related factors separately for men and women. All factors were included in the model. Variables were eliminated based on a Wald χ^2^ test for analysis of effect. Variables were removed one by one based on the significance level of their effect on the model, starting with the variable with the highest *P* value greater than 0.5, until all variables remaining had a value of *P* ≤ .50 in the analysis of effect. The logistic regression excluded cases with missing data. Of the 10,735 completed interviews, we excluded 398 observations that were missing obesity values, 141 missing blood pressure, 266 missing self-reported hypercholesterolemia status, 115 missing self-reported diabetes status, 32 missing self-reported chronic conditions, 212 missing fruits and vegetables consumption, 173 missing meat and chicken consumption, 29 missing smoking status, 33 missing marital status, and 20 missing educational level. In total, 10,293 observations were used in our regression analyses. Data were weighted to account for the probability of selection and age and sex poststratification based on census data for age and sex distribution of the Saudi population. We used SAS 9.3 (SAS Corp) for the analyses and to account for the complex sampling design.

## Results

From April through June 2013, we contacted a total of 12,000 households, and 10,735 participants completed the survey (response rate 89.4%).

Compared with male respondents, female respondents were younger, more likely to be married or previously married, less educated, and overweight or obese. Overall, 28.7%, or 3.6 million, Saudis aged 15 years or older were obese. This prevalence ranged from 24.1% among men to 33.5% among women (Table 1). Both men and women consumed low amounts of fruits and vegetables (more than 81.0% of men and women consumed fewer than 3 servings of fruits and vegetables per day) and most were physically inactive (46.0% of men, and 75.1% of women practiced low to no physical activity at all).

**Table 1:** Regression Analysis Predicting Distribution of Sociodemographic Characteristics and Body Mass Index Among Men and Women (N = 10,293)^a^, Kingdom of Saudi Arabia, 2013

Sociodemographic Characteristic	Men		Women		AOR (95% CI)
	N (Weighted %)	SE	N (Weighted %)	SE	
**Age, y**					
15–24	1,189 (40.8)	1.03	1,193 (39.8)	1.03	0.96 (0.96–0.97)
25–34	1,254 (21.7)	0.75	1,503 (21.3)	0.71	
35–44	1,132 (13.6)	0.53	1,207 (16.8)	0.62	
45–54	722 (12.0)	0.55	798 (12.8)	0.57	
55–64	439 (7.1)	0.44	423 (5.9)	0.39	
≥65	517 (4.9)	0.29	358 (3.5)	0.26	
**Marital status**					
Married	3,514 (49.3)	0.98	3,462 (49.4)	0.97	1 [Reference]
Never married	1,569 (49.3)	0.99	1,260 (42.4)	1.03	0.53 (0.46–0.63)
Separated, divorced, or widowed	159 (1.4)	0.15	738 (8.2)	0.17	6.64 (5.19–8.50)
**Education**					
Primary school or less	1,217 (20.3)	0.75	2,069 (32.5)	0.88	1 [Reference]
Elementary or high school completed	2,745 (59.3)	0.94	2,127 (46.2)	0.98	0.40 (0.34–0.48)
College degree or higher	1,282 (20.4)	0.72	1,275 (21.3)	0.77	0.55 (0.46–0.66)
**Body mass index, kg/m^2^**					
<18.5 (underweight)	238 (7.1)	0.59	209 (6.3)	0.55	1 [Reference]
18.5–24.9 (normal weight)	1,585 (35.4)	0.98	1,403 (32.3)	0.98	1.08 (0.81–1.43)
25.0–29.9 (overweight)	1,858 (33.4)	0.91	1,599 (28.0)	0.87	0.95 (0.71–1.26)
30.0–39.9 (obese)	1,273 (21.6)	0.78	1,767 (28.8)	0.85	1.52 (1.13–2.03)
≥40.0 (morbidly obese)	144 (2.5)	0.30	271 (4.7)	0.39	2.08 (1.38–3.13)

Abbreviations: SE, standard error; AOR, adjusted odds ratio; CI, confidence interval.

a Of the 10,735 participants surveyed, 10,293 had complete information and were included in this analysis.

Risk of obesity was lower among men who reported high levels of physical activity than among inactive men (Table 2). Men who were previously married, consumed 3 or more servings of meat per day, those who were previously diagnosed with diabetes or hypercholesterolemia, and those who had elevated blood pressure were more likely to be obese.

**Table 2:** Regression Analyses Predicting Obesity Risk in Relation to Sociodemographic Characteristics and Chronic Conditions Among Men and Women Aged 15 Years and Older, Kingdom of Saudi Arabia, 2013^a^

Sociodemographic Characteristic/ Risk Factor	Obese Men		Obese Women		Sociodemographic Model, OR (95% CI)		Backward Elimination Model, AOR (95% CI)	
	N (Weighted %)	SE	N (Weighted %)	SE	Men (N – 5,071)	Women (N = 5,222)	Men (N = 4,800)	Women (N = 4,950)
**Age^b^**	—b		—b		1.01 (1.01–1.02)	1.04 (1.03–1.04)	0.99 (0.98–1.00)	1.03 (1.02–1.03)
**Marital status**								
Married	1,136 (67.9)	2.01	1,456 (67.2)	1.56	—	—	1 [Reference]	
Never married	241 (30.8)	2.02	216 (21.2)	1.53	1.04 (0.61–1.78)	1.55 (1.09–2.22)	1.27 (0.69–2.34)	1.51 (1.03–2.23)
Separated, divorced, or widowed	38 (1.3)	0.26	360 (11.6)	0.82	1.99 (1.52–2.61)	2.14 (1.68–2.72)	2.07 (1.55–2.75)	2.32 (1.80–2.99)
**Education**								
Primary school or less	338 (22.0)	1.48	978 (45.8)	1.54	—	—	1 [Reference]	
Elementary or high school completed	684 (51.5)	1.88	659 (36.4)	1.50	1.10 (0.87–1.40)	0.87 (0.70–1.07)	1.00 (0.77–1.29)	0.91 (0.73–1.13)
College degree or higher education	393 (26.5)	1.61	396 (17.9)	1.15	1.45 (1.11–1.91)	0.74 (0.59–0.94)	1.31 (0.99–1.75)	0.77 (0.61–0.99)
**Tobacco smoking status**								
Never smoked	961 (67.9)	1.76	1,986 (97.5)	0.49	—	—	1 [Reference]	
Previous smoker	139 (8.6)	0.97	10 (0.4)	0.16	—	—	1.08 (0.78–1.49)	0.70 (0.22–2.21)
Current smoker	315 (23.5)	1.63	40 (2.1)	0.46	—	—	1.00 (0.79–1.27)	1.39 (0.78–2.49)
**Daily consumption of fruits and vegetables (servings)**								
0	34 (2.5)	0.61	54 (3.6)	0.60	—	—	1 [Reference]	
0.1–2.9	1,007 (73.6)	1.63	1,580 (76.4)	1.34	—	—	1.34 (0.74–2.42)	0.73 (0.45–1.19)
3. –4.9	212 (13.8)	1.22	240 (11.7)	1.01	—	—	1.73 (0.92–3.28)	0.76 (0.44–1.32)
≥5	141 (10.1)	1.12	135 (8.4)	0.90	—	—	1.80 (0.91–3.57)	0.85 (0.47–1.54)
**Daily consumption of red meat and chicken (servings)**								
0 – 0.9	313 (24.5)	1.61	719 (34.9)	1.45	—	—	1 [Reference]	
1 – 1.9	500 (36.9)	1.84	737 (36.9)	1.51	—	—	1.18 (0.93–1.51)	1.16 (0.95–1.43)
2 – 2.9	291 (15.4)	1.25	285 (12.6)	1.02	—	—	1.41 (1.05–1.88)	1.26 (0.94–1.68)
≥3	303 (23.1)	1.64	275 (15.5)	1.17	—	—	1.47 (1.10–1.98)	1.10 (0.83–1.45)
**Level of physical activity**								
None	470 (28.4)	1.61	1,046 (46.2)	1.52	—	—	1 [Reference]	
Insufficient	343 (25.6)	1.63	529 (29.7)	1.46	—	—	0.75 (0.58–0.96)	1.23 (0.99–1.51)
Moderate	234 (18.1)	1.52	182 (9.3)	0.94	—	—	0.79 (0.59–1.05)	1.30 (0.95–1.78)
Vigorous	370 (27.9)	1.74	281 (14.8)	1.11	—	—	0.54 (0.42–0.69)	1.12 (0.87–1.45)
**History of chronic condition diagnosis**								
No	1295 (90.9)	1.10	1,890 (91.8)	0.92	—	—	1 [Reference]	
Yes	121 (9.1)	1.10	145 (8.2)	0.92	—	—	1.24 (0.84–1.81)	1.80 (1.22–2.64)
**History of diabetes diagnosis**								
No	1,129 (82.3)	1.30	1,685 (87.3)	0.92	—	—	1 [Reference]	
Yes	280 (17.7)	1.30	334 (12.7)	0.92	—	—	1.46 (1.12–1.91)	1.05 (0.76–1.46)
**History of hypercholesterolemia diagnosis**								
No	1,177 (86.9)	1.16	1,782 (91.7)	0.78	—	—	1 [Reference]	
Yes	203 (13.1)	1.16	202 (8.3)	0.78	—	—	1.57 (1.16–2.14)	1.28 (0.86–1.91)
**Hypertension**								
Healthy	270 (20.0)	1.54	722 (38.8)	1.53	—	—	1 [Reference]	
Prehypertensive	655 (47.9)	1.89	780 (39.2)	1.51	—	—	1.89 (1.48–2.41)	1.64 (1.35–1.99)
Hypertensive	486 (32.2)	1.72	529 (22.1)	1.23	—	—	3.63 (2.70–4.88)	2.18 (1.65–2.89)

Abbreviations: SE, standard error; OR, odds ratio; CI, confidence interval; AOR, adjusted odds ratio; —,not included in the sociodemographic model.

a Models adjusted for sociodemographic characteristics: age, marital status, and education.

b Age was used as a continuous variable in the logistic regression models. Hence, the AOR for age indicates an increase of 1 year of age.

Among women, risk of obesity increased with age, being married or previously married compared with those never married, having been diagnosed with a chronic condition, and being prehypertensive or hypertensive (Table 2). Women who had more education than high school were less likely to be obese than those who had a primary school educational level or less. Diet indicators and physical activity were not significantly associated with obesity among women.

## Discussion

Our study indicated high rates of obesity across all segments of the population in KSA. Our findings showed that most Saudis are physically inactive and consume low levels of fruits and vegetables. Furthermore, we found strong associations between obesity and diabetes, hypercholesterolemia, and hypertension.

**Figure 2:**
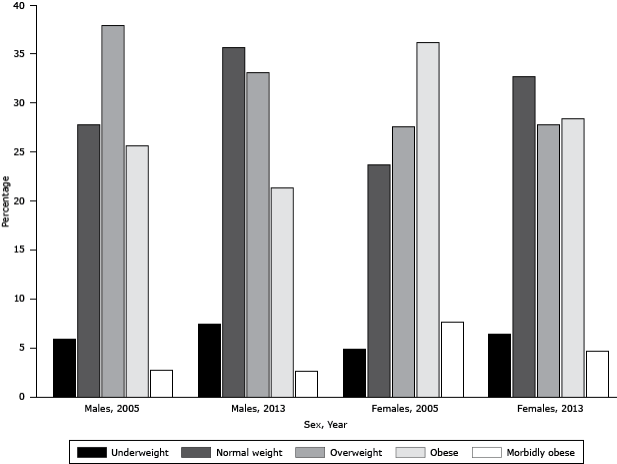
Percentage change in distribution of body mass index (BMI) categories from 2005 through 2013, for men and women, Kingdom of Saudi Arabia. Categories are underweight (BMI <18.5), normal weight (BMI 18.5–24.9), overweight (BMI 25.0–29.9), obese (BMI 30.0−39.9), or morbidly obese (BMI ≥ 40). **Table:** 

**Figure 3:**
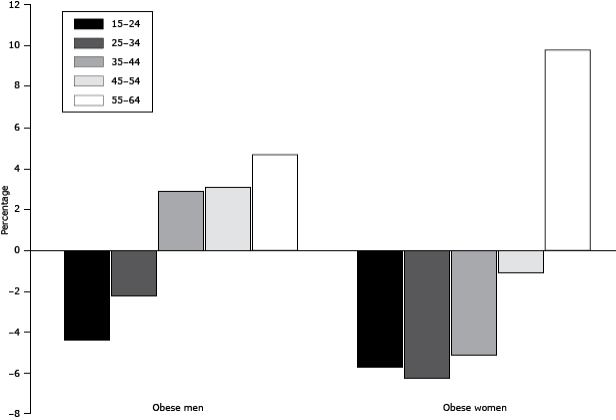
Percentage change in prevalence of obesity (body mass index [BMI] ≥30.0 kg/m^2^), by age group, for men and women, from 2005 through 2013, Kingdom of Saudi Arabia. **Table:** 

Obesity levels in our study are lower than those reported in 2005 (17) and those estimated for 2010 (18). Our survey had similar sampling frame and methods to the 2005 survey. When comparing our survey with the 2005 survey, we restricted the analyses to the same age groups. Obesity decreased by 4.4% for men and 10.7% for women between 2005 and 2013 (Figure 2). An increase of 7.8% for men and 9.0% for women in normal weight was observed during the same time period. A shift may have occurred from obese to overweight and from overweight to normal weight or from obese to normal weight. Conversely, the change in obesity prevalence differed significantly between age groups for both sexes (Figure 3). Whereas fewer men younger than 35 years were obese in 2013, older men had a higher prevalence of obesity. Obesity prevalence decreased for women of all age groups, except for those aged 55 to 64 years, among whom the prevalence of obesity increased by almost 10%. This overall decline in obesity prevalence is welcome news for the Saudis’ health, especially because there have been few success stories in decreasing obesity levels in other countries resulting from health interventions and environmental and policy changes (19,20). Over the last decade, the SMOH has implemented several public health programs to reduce obesity. Most of these programs have focused on awareness and behavioral changes (21). However, it is too early to determine whether the decline seen in this study is due to chance and thus cannot be sustained.

Sex/Year	Underweight	Normal Weight	Overweight	Obese	Morbidly Obese
Males, 2005	5.9	27.8	37.9	25.6	2.7
Males, 2013	7.4	35.6	33.1	21.3	2.6
Females, 2005,	4.9	23.7	27.6	36.2	7.6
Females, 2013	6.4	32.7	27.8	28.4	4.7

Sex	Age					
	15–24 y	25–34 y	35–44 y	45–54 y	55–64 y
Males	−4.4	−2.24	2.87	3.1	4.68
Females	−5.71	−6.26	−5.14	−1.11	9.8

Associations found in our study are similar to findings from previous studies, with varying results between men and women. The association between marital status and obesity has been previously cited (22). Despite being generally in better health than their counterparts, married men and women were found to be at higher risks of obesity than those who were unmarried. Among Saudis, risk of obesity was higher in married than in never-married men. Never-married women, as well as those who were separated, divorced, or widowed, were less likely to be obese than married women.

Obesity is higher among less-educated men and women (23). In the United States, as education levels increased, obesity levels declined among women but remained consistent among men. We observed the same pattern in KSA. With higher education, Saudi women may be making healthier choices that reflect on their body composition. Indeed, more educated women tend to improve their health profile and those of their children (24).

Smoking is associated with lower weight (25). We did not see this pattern in KSA. However, smoking rates among women in KSA were low. Similarly, fruit and vegetable consumption were not associated with obesity in our study. These findings differ from those reported elsewhere, because an increased daily consumption of fruits and vegetables is often associated with a decreased risk of obesity (26). Increased meat consumption, on the other hand, was associated with an increased risk of obesity among men. One explanation is that our participants might have misreported their fruit and vegetable intakes. Another explanation is that as Saudis increased their consumption of fruits and vegetables, they did not account for their total caloric intake.

Physical activity reduces the likelihood of obesity (27). A strong association between obesity and lack of physical activity has been shown among Saudi adolescents (28). This association was true among men, but not women, in our study. Whereas men were almost equally distributed between all levels of physical activity, most women reported being inactive or having a low level of physical activity.

Our findings about the lack of association between obesity and fruit and vegetable consumption and physical activity differ from those of previous studies. In KSA, the consumption of fruits and vegetables was similar among those who were obese and those who were not. This finding indicates that the patterns of fruit and vegetable consumption and physical activity are similar across women in KSA, making the effect of these factors undetectable in our analysis. A caloric imbalance, which we did not capture in our survey, may have been the driving factor of the epidemic.

The association we found between obesity and chronic noncommunicable diseases among Saudis is informative on the impact of obesity on chronic diseases in KSA. Diabetes, hypercholesterolemia, and elevated blood pressure were strongly associated with obesity in our study. With the declining rates of obesity over the last 8 years in KSA, we would expect an effect on these conditions in the Kingdom in the coming years. Although social disparities associated with obesity are less evident in KSA, and although the burden of obesity has decreased, its health consequences have not diminished. Saudis who had elevated blood pressure, hypercholesterolemia, or diabetes were more likely to be obese than healthier subjects. The global obesity epidemic has been strongly linked with these diseases (1). In 2010, hypertension was the second leading risk for death worldwide (29), and diabetes caused more than 1,200,000 deaths, compared with fewer than 600,000 in 1990 (29). Today, noncommunicable diseases are the major cause of death, illness, and disability. The success of controlling infectious diseases increased lifespans; however, this longer life expectancy comes at a cost. Specifically, in the Arab world, people live longer but not healthier lives (30). Hypertension and diabetes are contributing extensively to these unhealthy years lived with disability, increasing health expenditures and reducing quality of life.

Our study has some limitations. First, our data are cross-sectional, so we cannot assess causality in associations. Second, many of our behavioral data, such as diet and physical activity, are self-reported, and subject to recall and social desirability biases. Third, our dietary instrument was not designed to provide total caloric intake, so we were not able to control for the effect of specific food items while controlling for caloric intake. It also captures little information on bread and does not allow the assessment of grain consumption, which would have been an important nutrient to account for. Also, chronic conditions were self-reported, which leaves out respondents who might have these conditions but have not been previously diagnosed. Conversely, our study is based on a large sample size and used standardized methods for all its measures.

Although we focused our study on obesity as an extreme measure of excess weight, overweight should also be a target for prevention. Intervening with overweight Saudis before reaching the obesity level would be easier and more beneficial, especially if they have not yet developed adverse health events.

Our findings indicate that the epidemic of obesity in KSA may be leveling or decreasing. Obesity is a major risk factor for illness and death in KSA and has high associated health costs. Our findings point to the need for continued efforts to ensure a steady decline in obesity prevalence in KSA. For example, identified high risk groups, such as older, uneducated, and unmarried women, and unmarried and inactive men, should be targeted for obesity prevention programs. Saudis should be encouraged to follow a healthier diet by increasing consumption of fruits and vegetables and exercising.
